# P-2194. Evaluation of T cell-mediated immune response after dengue infection: a comparison between people living with HIV and HIV-negative individuals

**DOI:** 10.1093/ofid/ofaf695.2357

**Published:** 2026-01-11

**Authors:** Pierluigi Francesco Salvo, Francesca Lombardi, Gianmaria Baldin, Valeria Campolattano, Ilenia Aversa, Antonio Abatino, Simona Di Giambenedetto, Camillo Palmieri, Carlo Torti

**Affiliations:** Università Cattolica del Sacro Cuore, Roma, Lazio, Italy; Fondazione Policlinico A. Gemelli IRCCS, roma, Lazio, Italy; Fondazione Policlinico A. Gemelli IRCCS, roma, Lazio, Italy; Università Cattolica del Sacro Cuore, Roma, Lazio, Italy; Università Magna Grecia di Catanzaro, Catanzaro, Calabria, Italy; Università Magna Grecia di Catanzaro, Catanzaro, Calabria, Italy; Università Cattolica del Sacro Cuore, Roma, Lazio, Italy; Università Magna Grecia di Catanzaro, Catanzaro, Calabria, Italy; Università Cattolica del Sacro Cuore, Roma, Lazio, Italy

## Abstract

**Background:**

Dengue is the most widespread arboviral infection, with approximately half of the world's population at risk. In 2023, an autochthonous dengue outbreak occurred in Rome, highlighting the increasing relevance of this infection in non-endemic areas. PLWH are at an increased risk of developing severe dengue, particularly in cases of secondary infection. The aim of this study was to evaluate and compare the T cell-mediated immune response following dengue infection in PLWH and HIV-negative individuals.

**Methods:**

In this cross-sectional study, we enrolled PLWH and HIV-negative individuals with a history of dengue infection, identified through DENV RT-PCR or NS1 antigen assays in a hospital setting. IFN-γ enzyme-linked immunospot assay (ELISPOT) was used to analyze T cell response, using peptide pools representing three structural proteins (prM/E/C) and non-structural proteins (NS3 and NS2A/B, NS4A/B, NS5).

**Results:**

We enrolled 9 PLWH and 10 HIV-negative indiduals. All but one HIV-negative subjects showed a positive T-cell response to at least one stimulating peptide pool. Overall, HIV-negative individuals exhibited stronger ELISPOT responses than PLWH across all antigens, though without statistical significance, likely due to sample size limitations. The NS3 antigen showed the most pronounced difference (Mean: 23.5 in HIV-negative vs 2.0 in PLWH, p=0.223); prM/E/C responses were also higher in the HIV-negative group (14.3 in HIV-negative vs. 5.8 in PLWH, p = 0.327), while NS4/AB (5.4 in HIV-negative vs 2.5 in PLWH, p=0.451) and NS2/A/B (3.0 in HIV-negative vs 2.0 in PLWH, p =0.578) responses were only slightly elevated in the HIV-negative group. NS5 elicited the weakest response in both groups (0.5 in HIV-negative vs 0.0 in PLWH, p = 0.408).

**Conclusion:**

The non-uniform antigen recognition pattern between the 2 groups suggests potential differences in immune history, genetic background, or prior exposure between groups, influencing T-cell activation levels. Overall, our findings suggest that prior dengue exposure does not guarantee uniform T-cell reactivity, and HIV status may modulate the strength and pattern of antigen-specific immunity.

**Disclosures:**

All Authors: No reported disclosures

